# Germinated legumes for improving nutritional and technological quality of fortified cakes, with further enhancement using phospholipase and SSL

**DOI:** 10.1038/s41538-026-00819-2

**Published:** 2026-04-15

**Authors:** Shymaa M. Ata, Ahmed M. S. Hussein, Sayed Mostafa

**Affiliations:** 1 Home Economics Department, Faculty of Specific Education, Menoufia University, Shibin El Kom, Egypt; 2 Department of Food Technology, Food Industries and Nutrition Research Institute, National Research Centre, Cairo, Egypt

**Keywords:** Biochemistry, Biotechnology, Plant sciences

## Abstract

Bakery products represent a promising platform for functional food development. In this study, cakes were fortified with germinated chickpea flour (GCP) and germinated white kidney bean flour (GWKB) as partial substitutes for wheat flour (10, 20, and 30%), while phospholipase and sodium stearoyl lactylate (SSL) were applied to improve technological quality. The effects of germination on proximate composition, minerals, phenolic compounds, functional properties, protein digestibility, and antioxidant capacity were evaluated, in addition to pasting behavior, texture, and color attributes. Germination significantly enhanced water and oil absorption capacities, emulsifying and foaming properties, and increased protein digestibility, reaching 75.13% in GCP compared to 64.33% in raw chickpea. Post-storage crumb firmness was significantly reduced by phospholipase and SSL, with optimal freshness achieved at 75.45 ppm phospholipase and 0.6% SSL (R² = 95.74%). In conclusion, this study demonstrates a novel dual-enhancement strategy that integrates legume germination with combined enzymatic treatment and RSM-based optimization to simultaneously improve nutritional value and post-storage technological performance of fortified cakes, providing a practical model for the development of high-quality functional bakery products.

## Introduction

Cake is a widely consumed baked product worldwide, characterized by its soft texture and pleasant flavor, making it a popular choice among all age groups^1,2^. However, traditional cake, often prepared from whole wheat or white flour, is considered relatively low in nutritional value (lower protein and fiber), with a high glycemic index owing to its easily digestible carbohydrates^3^. This may not be suitable for some groups, such as diabetics or those seeking to enhance their nutritional status^4^.

Therefore, there has been increasing interest in fortifying cereal products, including cakes, bread, and pasta, with food sources high in fiber, protein, and bioactive substances, such as legumes, to improve the end product’s nutritional content and enhance its useful and healthful qualities^5,6^.

According to Summo et al.^7^, chickpea seeds (*Cicer arietinum* L.) are the most significant legume consumed worldwide, because of their greater dietary fiber and protein content (17.40–24.40%), in addition to being a good source of some minerals (iron, zinc, and magnesium), and amino acids like arginine and lysine^8,9^.

White kidney bean (*Phaseolus vulgaris* L.) is an important legume, characterized by their high protein content (21.70–27.30%) and 11.40–33.30% of non-starch carbohydrates^10^.

However, the germination process of legume seeds is an effective method that many studies have shown to be beneficial in enhancing its biological and nutritional value^11^. It also contributes to the reduction of tannins, phytic acid, and digestive enzyme-inhibiting compounds^12^, which increases the availability of Zn and Fe^13^ and improves the digestion of protein^14,15^.

Overall, although legume fortification substantially enhances the nutritional quality of bakery products, it often compromises their technological performance, particularly by accelerating staling and increasing crumb firmness. This technological drawback can be mitigated through the use of functional improvers, such as chemical emulsifiers or enzymatic treatments, which help bridge the quality gap and enable the development of products that combine superior nutritional value with desirable technological attributes. In this context, enzymatic treatments have proven particularly effective in improving multiple quality parameters of baked goods, such as texture and specific volume^16^, and have also shown potential in supporting gluten-free bakery applications through gluten degradation^17^. Phospholipases, for example, function as anti-staling agents by hydrolyzing flour phospholipids into lysophospholipids with high surface activity, which interact with starch and proteins to stabilize gas cells and enhance crumb softness. These compounds interact with gluten and starch molecules, enhancing the stability of gas cells and leading to improve the crumb softness^18^. Similarly, sodium stearoyl lactylate (SSL) acts as an emulsifier that contributes to dough strengthening by forming complexes with gluten proteins and starch amylose, reducing starch retrogradation during storage^19^. However, recent in-vitro studies^20,21^ have reported that dietary emulsifiers such as SSL may disturb gut microbiota composition, reduce beneficial butyrate-producing bacteria, and potentially contribute to low-grade intestinal inflammation; therefore, its use should be maintained at levels permitted for food applications. Unlike previous studies that examined either germination alone^22^, single-legume fortification^23^, or enzymatic improvers of wheat products^24^, the present study uniquely integrates dual-legume germination, combined enzymatic enhancement using phospholipase and SSL, and an RSM-based optimization model to improve both the nutritional and technological quality of fortified cakes.

The novelty of the present study resides in addressing the persistent technological drawbacks associated with legume fortification in cake systems through a combined strategy that links controlled dual-legume germination with enzymatic improvement and statistical optimization. By integrating these components within a single experimental framework, this work moves beyond isolated nutritional or technological modifications and establishes a coordinated approach to achieving balanced quality enhancement in fortified cakes.

Accordingly, the purpose of the current study is to evaluate the impact of the germination process on the physicochemical characteristics of chickpeas and white kidney beans, in addition to producing a cake enriched with germinated chickpea flour (GCP) and germinated white kidney bean flour (GWKB) to enhance the nutritional quality of the cake as a final product. The study also evaluated the impact of phospholipase and SSL on improving cake freshness and quality across different substitution ratios.

## Results and discussion

### Chemical characteristics of germinated and raw legume flour

The effect of germination on the approximate analysis and mineral content (on dwb) is shown in Table 1. Ash content was significantly (*p* < 0.05) lower in germinated chickpea (GCP: 3.17%) compared to raw chickpea (RCP: 3.35%), while the difference was not significant between raw white kidney bean (RWKB) and GWKB regarding the ash content. On the other hand, the germination process showed a positive impact on protein content for the used legumes, with a significant increase compared to raw legumes. The increase in protein is often due to the decrease in carbohydrate content after germination as a source of energy and embryo growth^25^. This also reflects the effect of germination in stimulating enzyme activity and decomposing complex compounds, which increases the protein percentage and protein bioavailability^26^. The protein content of CP, beans, and lentils increased significantly after soaking and germination, with increased activity of converting enzymes such as transaminases^27^. The fat content also decreased significantly after germination in both white kidney beans and CP, possibly due to the utilization of lipids as an energy source during germination^28^. Data also indicated no significant changes in the crude fiber content of CP before and after germination (5.55 and 5.38%, respectively), while the crude fiber content decreased significantly in GWKB (3.36%) compared to RWKB (3.65%), with a significant significance. Finally, the nitrogen free extract (NFE) significantly decreased after germination in both CP and white kidney beans, that reflect the consumption of sugars during germination^11^.

**Table 1:** Chemical characteristics and minerals content of germinated and raw legume flour

Chemical parameters (on dwb)	Samples				*p*-values	LSD at 0.05
	RCP	GCP	RWKB	GWKB		
**Ash (%)**	3.35^a^ ± 0.04	3.17^b^ ± 0.01	3.13^b^ ± 0.02	3.24^ab^ ± 0.04	<0.0001	0.084
**Protein (%)**	20.10^c^ ± 0.10	22.25^a^ ± 0.09	18.49 ^d^ ± 0.23	21.08^b^ ± 0.05	<0.0001	0.765
**Fat (%)**	4.37^a^ ± 0.06	4.03^b^ ± 0.03	1.86^c^ ± 0.06	1.64 ^d^ ± 0.04	<0.0001	0.182
**CF (%)**	5.38^a^ ± 0.09	5.55^a^ ± 0.06	3.65^b^ ± 0.01	3.36^c^ ± 0.09	<0.0001	0.214
**NFE (%)**	66.81^c^ ± 0.08	65.01 ^d^ ± 0.19	72.88^a^ ± 0.18	70.70^b^ ± 0.03	<0.0001	1.523
**Fe (mg/100** **g)**	6.11^a^ ± 0.20	5.87^a^ ± 0.11	3.42^b^ ± 0.02	3.01^c^ ± 0.03	<0.0001	0.329
**Zn (mg/100** **g)**	3.03^a^ ± 0.02	2.89^b^ ± 0.03	1.33^b^ ± 0.02	1.15^c^ ± 0.01	<0.0001	0.071
**K (mg/100** **g)**	733.90^a^ ± 10.54	718.02^a^ ± 13.77	463.63^b^ ± 12.87	411.00^c^ ± 5.89	<0.0001	16.936
**Mg (mg/100** **g)**	111.30^a^ ± 5.67	100.34^a^ ± 2.28	81.21^b^ ± 2.17	88.24^b^ ± 3.24	<0.0001	11.834
**Ca (mg/100** **g)**	63.67^a^ ± 1.66	60.23^a^ ± 1.13	32.54^b^ ± 0.96	26.16^c^ ± 1.02	<0.0001	3.878

Values from triplicate determinations are presented as means ± SD (*n* = 3). Different superscript letters (a, b, c, etc.) within the same column indicate significant differences (*p* < 0.05).

*CF* crude fiber, *NFE* nitrogen free extract, *RCP* raw chickpea, *GCP* germinated chickpea, *RWKB* raw white kidney bean, *GWKB* germinated white kidney bean.

As for the effect of germination on the mineral content of CP. The results indicate that germination does not lead to significant changes in the content of most minerals in CP, with the exception of zinc, which was significantly decreased after germination. This may be attributed to its loss during the soaking and germination processes or its association with non-absorbable compounds.

Regarding white beans, the iron content decreased from 3.42 to 3.01 mg/100 g, and zinc from 1.33 to 1.15 mg/100 g, with significant differences. However, magnesium content increased from 81.21 to 88.24 mg/100 g, but without significant differences, suggesting that this element may be more stable during processing or perhaps undergoes a relative release from its associated compounds during germination. These results reflect that germination significantly affects most of the mineral elements in white beans, especially iron, zinc, potassium, and calcium. Atudorei et al.^22^, observed a decrease in iron content in soy beans, CP, and lupine until the fourth day of germination. However, an increase in iron content may be observed in beans and lentils on the fourth day of germination. Zinc and manganese content may also increase or decrease, depending on the legume variety. Others^29^ stated that the germination process led to decrease the iron content of faba bean (2.45 mg/100 g to 2.38 mg/100 g), and soybean (26.80 mg/100 g to 25.70 mg/100 g).

### Functional properties

**Fig. 1:**
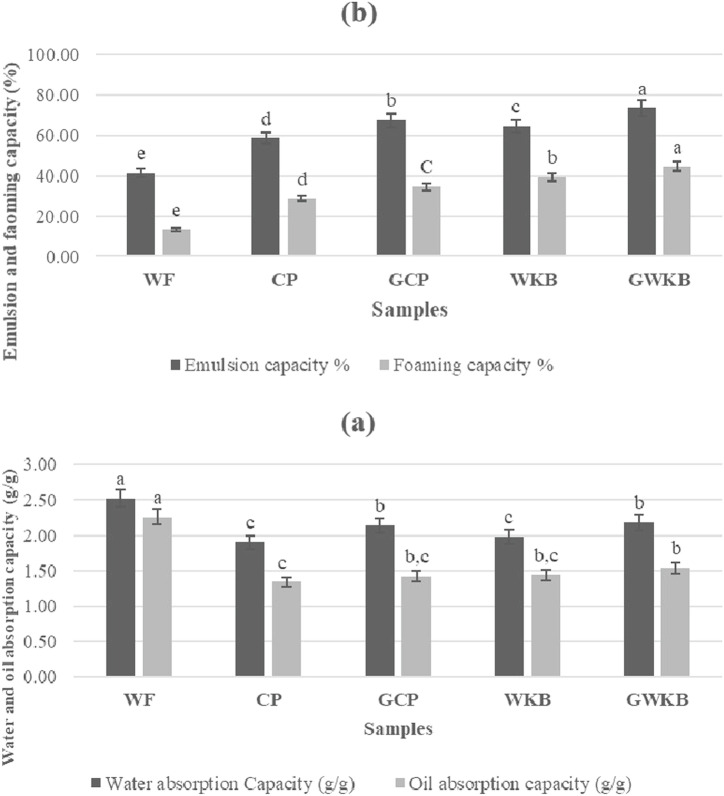
Functional properties of wheat flour compared to raw and germinated legume flours. **a** Water and oil absorption capacities; **b** emulsion and foaming capacities. Bars with different superscript letters (a, b, c, etc.) indicate significant differences between samples (*p* < 0.05).

Figure 1a, b illustrated the functional properties of wheat flour and the effect of germination of chickpea and white kidney beans on the functional properties. Wheat flour (WF) significantly recorded the highest values (2.53 g/g and 2.26 g/g) of water absorption capacity (WAC) and oil absorption capacity (OAC), respectively, compared to both germinated and ungerminated CP or white beans, due to its rich composition of gluten and water- and oil-binding proteins^30^. Germination showed a significant increase in WAC for both CP and beans, while no significant changes in OAC values after germination, indicating an increase in the ability of modified proteins and carbohydrates to bind to water molecules, possibly as a result of partial breakdown of the cell walls during germination.

Data also indicated, wheat flour significantly recorded the lowest values for both emulsion capacity (41.23%) and foaming capacity (13.35%). A significant increase in emulsion capacity for CP and white kidney beans was observed after germination, reflecting an improvement in the surface activity of proteins due to structural changes associated with germination^31^. In addition, there is a positive effect of germination on foaming capacity, which indicates an improved air-trapping ability of the germinated proteins, which is associated with increased solubility and flowability after germination^32,33^. Overall, these results show that germination clearly improves the functional properties of CP, which enhances their potential for use in diverse food applications^34^.

As reported by Elbaum and Abraham^35^, structural changes in the proteins and sugars after germination make them more capable of retaining water as a result of the collapse of some cell walls and increased porosity. The increase in foaming capacity indicates an improvement of the surface proteins responsible for air trapping and foam stabilization after germination. These indicate the germination significantly enhances the functional properties of legumes by increasing their suitability for use in food products that require wetting, emulsifying, or foaming properties^36^.

### In vitro protein digestibility

**Fig. 2:**
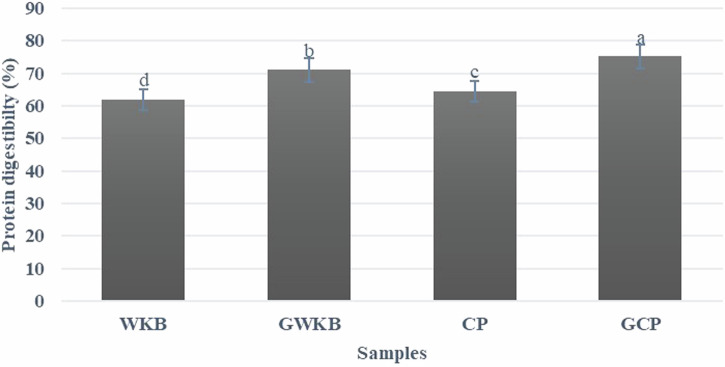
In vitro protein digestibility of germinated and ungerminated legumes. Bars with different superscript letters (a, b, c, etc.) indicate significant differences between samples (*p* < 0.05).

As shown in Fig. 2, the germination process significantly increased in vitro protein digestibility (IVPD) in both CP and WKB, where the IVPD was increased from 61.94 to 70.99% after germination of WKB with significant differences (*p* < 0.05). Meanwhile, the CP showed a significant increase in IVPD after germination, recording 64.33% for raw CP up to 75.14% for GCP. This improvement reflects the effect of germination in reducing anti-nutritional compounds (trypsin inhibitors and phytic acid), as well as activating endogenous enzymes that lead to decomposition of complex protein into simple form, more digestible units. These results reflect the positive role of germination in improving the nutritional value of legumes by enhancing protein digestion efficiency. Germination is a natural biological process that activates endogenous enzymes in legume seeds, such as protease and phytase^15^, this degrades stored proteins into amino acids and simpler peptides that are easily digestible by human digestive enzymes^37^. Germination also significantly reduces the phytic acid, tannin, and trypsin inhibitors, which directly inhibit digestive enzymes, enhancing protein absorption^38,39^. Laboratory studies stated that sprouting results in an increase in the in vitro protein digestibility rate of legumes (up to 4.40%) such as CP, lentils, and beans^40^. Germination enhances the structural properties of proteins by increasing solubility and reducing molecular weight, facilitating better digestion^41,42^. These results provide practical evidence that sprouting increases protein content and enhances its bioavailability and digestibility, making legumes a more effective plant source of protein.

### Anti-nutritional factors of raw and germinated legume flour

**Fig. 3:**
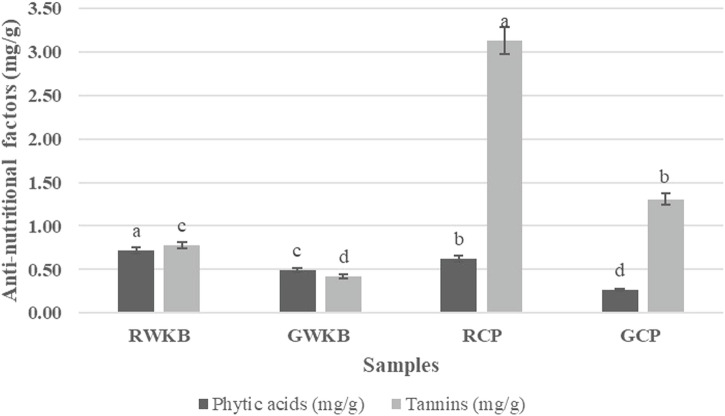
Anti-nutritional factors of germinated and ungerminated legumes. Bars with different superscript letters (a, b, c, etc.) indicate significant differences between samples (*p* < 0.05).

The effect of germination on the levels of phytic acid and tannins in CP and WKB is presented in Fig. 3. Concerning WKB, the phytic acid significantly (*p* < 0.05) decreased from 0.72 to 0.49 mg/g, and tannins also decreased from 0.78 to 0.42 mg/g after germination with significant differences. Likewise, the phytic acid content of raw CP (0.63 mg/g) significantly decreased to 0.27 mg/g after germination. Also, the tannin content significantly decreased from 3.13 to 1.31 mg/g for raw and germinated chickpea, respectively. This significant decrease shows the effective role of germination in degrading or reducing compounds that hinder nutrient absorption and protein digestion^43,44^. This is directly related to previous findings (Fig. 2), which showed improved protein digestibility after germination, as these compounds are factors that hinder the effectiveness of digestive enzymes or form indigestible complexes with proteins. Therefore, reducing their levels directly contributes to improving digestion efficiency and increasing the nutritional benefit of plant proteins^45,46^. Phytic acid and proteins combine to produce complexes, reducing the activity of the enzyme responsible for protein digestion^47^. Phytate anions can bind indirectly to negatively charged protein functional groups or directly to positively charged protein functional groups via a multivalent cation bridge^48^.

### Total phenolic and antioxidant activity

**Fig. 4:**
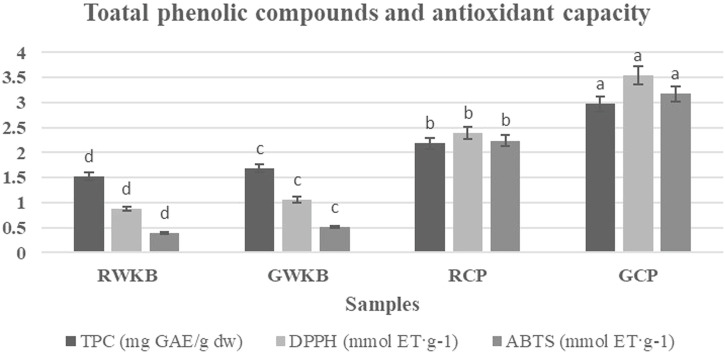
Total phenolic compounds (TPC) and antioxidant capacity of germinated and ungerminated legumes. Bars with different superscript letters (a, b, c, etc.) indicate significant differences between samples (*p* < 0.05).

The effect of germination on the total phenolic compounds (TPC) content and antioxidant capacity (DPPH and ABTS) of both CP and WKB was estimated, and the obtained results are shown in Fig. 4. The TPC significantly increased from 2.18 to 2.97 mg GAE/g after germination of CP (*p* < 0.05) followed by a significant increase in DPPH and ABTS capacity (3.54% and 3.17 mmol ET·g-1, respectively) compared to the lower values recorded by raw CP (2.39 and 2.24%, respectively). The same trend was noted in the case of WKB before and after germination, with significant differences. These results suggest that germination may contribute to modifying chickpea components in a way that retains a significant portion of the antioxidant capacity. These findings are consistent with Umar et al.^49^, they explained that the process of sprouting legumes has an effective role in improving the level of phenolic compounds and antioxidants, where the phenolic content of lentils and germinated beans was higher, and that of ungerminated beans was much lower. The germination led to a boost in nutritious components and antioxidants in beans and lentils, and the resulting beans were a great source of natural antioxidants. On the contrary, Dicko et al.^50^, reported that phenolic compounds were decreased after germination of sorghum varieties, which in turn negatively affected the antioxidant activity compared to ungerminated grains. Germination stimulates the activity of phenylalanine-ammonia-lyase, leading to increased synthesis of phenolic compounds and flavonoids in legumes^51,52^, thereby significantly enhancing their antioxidant capacity^34^. Germination also releases cell wall-bound phenolic compounds, increasing their bioavailability and biological activity^53^. Studies on CP and lupine have shown an increase in total phenolics and antioxidant activity after germination^54^.

### Pasting behavior of the wheat flour and the different legume flours

**Fig. 5:**
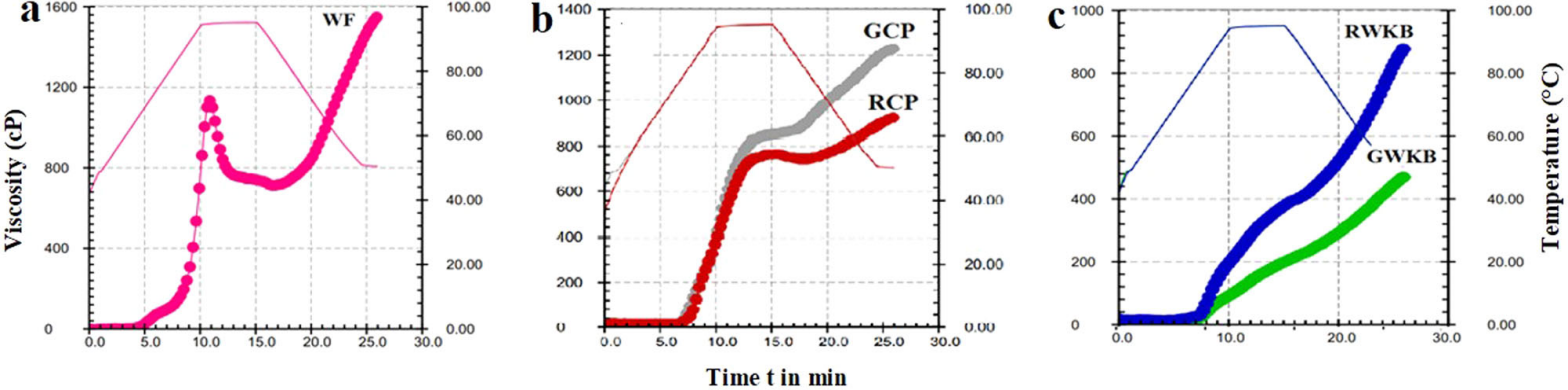
Pasting behavior curves of wheat and legume flours. **a** Wheat flour; **b** raw chickpea flour and germinated chickpea flour; **c** raw white kidney beans flour and germinated white kidney beans flour.

The pasting behavior of starches is tracking viscosity and simulating the cooking conditions, including variations during heating and cooling. According to the presented data in Table 2 and Fig. 5a–c, the germination showed a significant increase (*p* < 0.05) in the peak viscosity (PV), which rose from 764.8 to 850.3 cP in germinated CP, indicating an improved ability of starch granules to swell and retain water after germination, likely due to a decrease in swelling inhibitors^55^. Higher viscosity suggests more water access to granules, which may be brought about by a weakening of the protein and fiber matrix as germination proceeds, according to Setia et al.^56^. This increase was also accompanied by increases in both final viscosity (FV) and setback viscosity (ST), suggesting a greater ability to form a strong and stable starch gel after cooling. While wheat flour remained at the highest PV (1134 cP), FV, and ST values, suggesting its high swelling starch content and the absence of the amylase inhibitors found in legumes^57^. In addition, the viscosity peak temperature (PT) increased from 60.1 to 69.0 °C after chickpea germination, indicating a structural change in the starch or greater interference from its associated proteins. For germinated white kidney beans, PV significantly decreased from 377.1 to 197.8 cP, followed by decreases in FV and ST, supporting the idea that sprouting enhances the pasting properties of the legume, although these values were still low compared to CP and wheat flour^58^.

**Table 2:** Pasting behavior

Pasting parameters	Samples					*p*-value	LSD at 0.05
	WF	Legumes					
		RCP	GCP	RWKB	GWKB		
PV (cP)	1134^a^ ± 17.0	764.80^c^ ± 8.3	850.30^b^ ± 5.2	377.10 ^d^ ± 9.1	197.80^e^ ± 3.6	<0.0001	22.564
T (min)	10.80^b^ ± 0.1	14.80^a^ ± 0.2	15.00^a^ ± 0.4	15.00^a^ ± 0.1	15.00^a^ ± 0.3	<0.0001	0.652
PT (°C)	58.00 ^d^ ± 0.8	60.10^c^ ± 0.4	69.00^a^ ± 0.7	63.50^b^ ± 0.4	69.70^a^ ± 0.2	<0.0001	1.309
Peak T. (°C)	94.70^a^ ± 0.8	95.10^a^ ± 0.6	95.10^a^ ± 0.4	95.10^a^ ± 0.8	95.10^a^ ± 0.4	<0.0001	1.225
HS (cP)	709.90^c^ ± 9.5	744.90^b^ ± 8.0	849.90^a^ ± 7.6	377.10 ^d^ ± 11.3	197.80^e^ ± 5.2	<0.0001	11.842
BD (cP)	423.90 ± 8.1	19.85 ± 1.5	0.314 ± 0.01	0.00 ± 0.00	0.00 ± 0.00	-	-
FV (cP)	1554.00^a^ ± 27.1	923.70^c^ ± 13.6	1231.00^b^ ± 19.2	883.00 ^d^ ± 11.0	471.60^e^ ± 9.4	<0.0001	33.853
ST(cP)	1130.00^b^ ± 19.7	903.80^c^ ± 22.0	1230.00^a^ ± 9.5	883.00 ^d^ ± 14.0	471.60^e^ ± 16.3	<0.0001	27.847

Values from triplicate determinations are presented as means ± SD (*n* = 2). Different superscript letters (a, b, c, etc.) within the same row indicate significant differences (*p* < 0.05).

*PV* peak viscosity, *PT* pasting temperature, *T* peak time, *HS* holding strength, *Peak T* peak temperature, *BD* breakdown, *ST* setback from trough, *FV* final viscosity, *WF* wheat flour, *RCP* raw chickpea, *GCP* germinated chickpea, *RWKB* raw white kidney bean, *GWKB* germinated white kidney bean.

### Amino acids quantification of raw and geminated legume flour

Germination had different effects on CP and WKB. As shown in Table 3, it was noted that germination led to a slight relative increase (RI) in histidine content in CP after germination, amounting to 1.92%. While histidine content decreased in WKB after germination, amounting to −8.33%. A decrease in sulfur amino acids was also observed in both CP and WKB after germination. In contrast, tyrosine and phenylalanine were increased after germination of CP and WKB. The results also showed an increase in lysine content in CP after germination, amounting to a relative increase of 24.11%, compared to a 17.24% relative increase observed in WKB after germination. The highest relative increase in valine (45.02%) was observed in CP after germination, while WKB recorded the highest relative increase for leucine after germination (16.85%). Regarding the effect of germination on the proportion of non-essential amino acids, a slight increase was noted in aspartic acid, glutamic acid, and alanine in CP and WKB, while germination led to a relative decrease in serine, glycine, arginine, and proline in CP after germination. The highest percentage of decrease was observed in glycine (−25.61%) and proline (−11.87%) in WKB after germination. Regarding the protein quality, germination led to a relative increase in the essential amino acids of CP and WKB by 14.46% and 14.35%, respectively. Both CP and WKB also recorded a relative increase in branched-chain amino acids after germination (23.60% and 21.04%, respectively). However, WKB was more affected than CP in terms of C-PER and BV, with the highest relative increase for each of them being recorded with WKB (26.92% and 7.03%, respectively). Therefore, the germination process is an effective means of improving the nutritional value of legumes, both in terms of the quantity and quality of amino acid content, which enhances their potential for use in functional and supplementary food products. Generally, legume germination brings about fundamental changes in the protein and amino acid profile of legumes due to the proteolytic enzyme activity (proteases) and endopeptidases and exopeptidases, which degrade stored proteins into free peptides and easily absorbable amino acids^15,42,59^. During this process, the content of essential amino acids increases, especially branched-chain amino acids^60^. The developing embryo relies on these amino acids as a source of energy and to support the synthesis of new growth-related proteins, stimulating their formation or release from storage proteins^61^.

**Table 3:** Amino acids profile

Amino acids (g/100 g protein)	Samples					
	RCP	GCP	RI (%)	RWKB	GWKB	RI (%)
	Essential amino acids					
Histidine	3.12	3.18	1.92	2.16	1.98	−8.33
Threonine	3.96	3.91	−1.26	3.82	3.93	2.88
Tyrosine	2.44	3.03	24.18	2.76	3.64	31.88
Valine	4.22	6.12	45.02	5.28	6.30	19.32
Methionine	1.11	0.90	−18.92	0.92	0.80	−13.04
Cysteine	0.62	0.56	−9.68	0.50	0.43	−14.00
Isoleucine	3.35	4.58	36.72	3.97	5.13	29.22
Leucine	7.98	8.52	6.77	5.58	6.52	16.85
Phenylalanine	4.96	5.23	5.44	4.57	4.97	8.75
Lysine	3.36	4.17	24.11	3.48	4.08	17.24
Non-essential amino acids						
Aspartic acid	11.64	11.66	0.17	11.95	12.22	2.26
Glutamic acid	18.17	18.37	1.10	17.87	18.22	1.96
Serine	4.36	4.24	−2.75	3.32	3.37	1.51
Glycine	4.05	3.21	−20.74	6.60	4.91	−25.61
Arginine	10.96	9.63	−12.14	8.24	7.76	−5.83
Alanine	4.39	4.51	2.73	6.27	6.45	2.87
Proline	4.12	3.67	−10.92	3.96	3.49	−11.87
Protein quality						
TEAAs	35.12	40.20	14.46	33.04	37.78	14.35
TNEAAs	57.69	55.29	−4.16	58.21	56.42	−3.08
TAAs	92.81	95.49	2.89	91.25	94.20	3.23
TAAAs	7.40	8.26	11.62	7.33	8.61	17.46
TBCAAs	15.55	19.22	23.60	14.83	17.95	21.04
Fischer ratio (%)	2.10	2.33	10.73	2.02	2.08	3.04
C-PER	2.76	3.03	9.68	1.67	2.13	26.92
BV (%)	78.98	81.79	3.57	67.53	72.28	7.03

*RCP* raw chickpea, *GCP* germinated chickpea, *RWKB* raw white kidney bean, *GWKB* germinated white kidney bean, *RI* relative increase, *TEAAs* total essential amino acids, *TNEAAs* total non-essential amino acids, *TAAs* total amino acids, *TAAAs* total aromatic amino acids, *TBCAAs* total branched chain amino acids, *C-PER* calculated protein efficiency ratio, *BV* biological value.

### Chemical characteristics of wheat flour cake and legume-fortified cakes

As presented in Table 4. The control cake (100% wheat flour) significantly (*p* < 0.05) showed the lowest moisture (24.63%) and protein contents (9.30% dwb). A gradual increase in moisture content was observed with the increase of replacement level of GCP or GWKB, with the sample containing 30% GWKB recording the highest moisture content (28.53%), with a significant difference compared to the other samples. The control sample had the lowest ash content, with no significant changes compared to the samples containing 10% of either GCP or GWKB. The protein content of the cake increased proportionally with the percentage of germinated legume flour, with the highest protein content (13.88% dwb) recorded by the sample containing 30% GCP flour, followed by the sample containing 30% GWKB (13.32% dwb), with a significant difference between them. No significant changes were observed between the cake samples in terms of fat content, while the control sample recorded the highest content of NFE. Crude fiber content also gradually increased, achieving significant differences at all substitution ratios compared to the control sample, except the sample containing 10% GCP. Overall, GCP and GWKB improved the nutritional value of the cake compared to the control regarding mineral content, noting that GCP showed greater superiority in raising protein content, while GWKB significantly increased moisture and fiber content. Numerous studies are consistent with these findings, indicating that fortifying cereal products with legumes improves nutritional value through higher protein, mineral, and fiber content and lower NFE content compared to products made from wheat flour^62,63^.

**Table 4:** Chemical characteristics of cake samples

Cake samples	Moisture	Approximate analysis (% on dwb)				
		Ash	Protein	Fat	Crude fiber	NFE
Control	24.63 ^d^ ± 0.04	0.82^c^ ± 0.04	9.30 ^g^ ± 0.11	23.15^b^ ± 0.08	0.12^e^ ± 0.01	66.61^a^ ± 0.23
S1	25.48^c^ ± 0.21	0.97^bc^ ± 0.04	11.24^e^ ± 0.09	23.27^ab^ ± 0.05	0.24^de^ ± 0.01	64.29^b^ ± 0.20
S2	27.10^b^ ± 0.01	1.07^ab^ ± 0.05	12.41^c^ ± 0.05	23.30^ab^ ± 0.05	0.38^bc^ ± 0.04	62.86^c^ ± 0.19
S3	27.77^b^ ± 0.33	1.17^a^ ± 0.05	13.88^a^ ± 0.03	23.36^ab^ ± 0.10	0.49^a^b ± 0.03	61.11 ^d^ ± 0.21
S4	25.99^c^ ± 0.02	0.97^bc^ ± 0.04	10.76 ^f^ ± 0.08	23.47^ab^ ± 0.18	0.28 ^cd^ ± 0.02	64.51^b^ ± 0.32
S5	27.47^b^ ± 0.06	1.06^ab^ ± 0.04	12.07 ^d^ ± 0.05	23.58^ab^ ± 0.15	0.46^b^ ± 0.05	62.82^c^ ± 0.28
S6	28.53^a^ ± 0.26	1.15^a^ ± 0.04	13.32^b^ ± 0.06	23.70^a^ ± 0.13	0.61^a^ ± 0.03	61.21 ^d^ ± 0.26
*p*-value	<0.0001	0.0009	<0.0001	0.0208	<0.0001	<0.0001
LSD at 0.05	0.704	0.169	0.284	0.462	0.122	0.966

Values from triplicate determinations are presented as means ± SD (*n* = 3). Different superscript letters (a, b, c, etc.) within the same column indicate significant differences (*p* < 0.05). The cake samples were formulated using wheat flour partially substituted with germinated legume flours. Chickpea flour, used in S1-S3 at substitution levels of 10%, 20%, and 30%, respectively, while white kidney bean flour, used in S4-S6 at the same levels, respectively. The control sample contained 100% wheat flour.

*NFE* nitrogen free extract.

### Crust color attributes of wheat flour cake and legume-fortified cakes

The crust color of the control cake (100% wheat) and other legume-fortified cakes was measured (Table 5). The obtained data stated that the L* (lightness) value significantly decreased with increasing levels of GCP in the prepared cake compared to the control cake, indicating a darker crust color. Meanwhile, the a* (color tendency toward redness) value gradually increased with increasing levels of GCP, recording significant differences at all substitution ratios compared to the control. The b* (color tendency toward yellowness) value also increased significantly from 27.82 in the control to 31.31 in the sample containing 30% GCP. As a result of these changes, the chroma index (Chroma) increased and remained at high levels (70.29–71.57) in GCP-fortified cakes, while the browning index (BI) increased significantly from 66.76 in the control to 88.75 in the 30% GCP, clearly demonstrating the effect of melanoidins produced by the Maillard reaction during baking. Conversely, the whiteness index (WI) decreased significantly from 52.99 (control) to 46.14 (30% GCP), indicating a loss of whiteness in favor of a darker, browner crust. According to the statistical data, no significant changes between the control sample and GWKB-fortified cake in terms of yellowness (b* values) and hue at all replacement levels. While the lightness values significantly decreased compared to the control cake. Conversely, the redness values were significantly increased with the increasing level of GWKB.

**Table 5:** Crust color attributes of cake samples

Samples	Crust color parameters						
	L*	a*	b*	Chroma	Hue	BI	WI
**Control**	63.32^a^ ± 0.03	9.46 ^d^ ± 0.11	27.82^c^ ± 0.37	71.21^a^ ± 0.04	29.38^a^ ± 0.38	66.76 ^d^ ± 1.05	52.99^a^ ± 0.22
**S1**	61.11^bc^ ± 0.16	10.15^c^ ± 0.21	30.46^ab^ ± 0.48	71.57^a^ ± 0.09	32.10^abc^ ± 0.52	78.25^bc^ ± 1.95	49.57^bc^ ± 0.46
**S2**	59.85 ^d^ ± 0.21	10.90^b^ ± 0.14	31.09^a^ ± 0.01	70.68^a^ ± 0.24	32.95^ab^ ± 0.04	83.32^ab^ ± 0.55	48.06^c^ ± 0.19
**S3**	57.64^e^ ± 0.51	11.21^b^ ± 0.12	31.31^a^ ± 0.18	70.29^ab^ ± 0.30	33.26^a^ ± 0.13	88.75^a^ ± 1.49	46.14 ^d^ ± 0.48
**S4**	62.15^b^ ± 0.21	10.30^c^ ± 0.14	29.20^bc^ ± 0.85	70.56^a^ ± 0.77	30.96^c^ ± 0.75	73.17^c^ ± 2.52	51.09^b^ ± 0.64
**S5**	61.22^bc^ ± 0.31	11.05^b^ ± 0.21	28.95^bc^ ± 0.07	69.10^bc^ ± 0.32	30.98^c^ ± 0.14	74.78^c^ ± 0.03	50.35^b^ ± 0.15
**S6**	60.75 ^cd^ ± 0.35	11.94^a^ ± 0.06	29.32^bc^ ± 0.11	67.84^c^ ± 0.17	31.66^bc^ ± 0.08	77.69^bc^ ± 0.84	49.57^bc^ ± 0.33
***p*-value**	<0.0001	<0.0001	0.0003	<0.0001	<0.0001	<0.0001	<0.0001
LSD at 0.05	1.155	0.595	1.592	1.399	1.517	5.961	1.541

Values from triplicate determinations are presented as means ± SD (*n* = 3). Different superscript letters (a, b, c, etc.) within the same column indicate significant differences (*p* < 0.05). The cake samples were formulated using wheat flour partially substituted with germinated legume flours. Chickpea flour was used in S1–S3 at substitution levels of 10%, 20%, and 30%, respectively, while white kidney bean flour was used in S4-S6 at the same levels, respectively. The control sample contained 100% wheat flour. L*, a* and b* represent the CIELAB color parameters (lightness, redness and yellowness, respectively).

*BI* browning index, *WI* whiteness index.

Replacing a portion of wheat flour with chickpea or bean flour results in a lower L-value (lightness or brightness), resulting in a relatively darker crust color due to the higher protein and reducing sugar content of the legumes, which enhances the Maillard reaction during baking^64^. The crust also exhibits increased yellowness (higher b-value) due to the natural color of legumes, especially GCP, which are rich in carotenoid pigments, as well as the formation of yellow-brown color during baking. Furthermore, the high fiber and mineral content of legumes enhances pigment stability and color intensification, giving the crust a darker yellow-brown appearance compared to traditional cakes made solely from wheat^65–68^.

### Quality attributes of wheat flour cake and legume-fortified cakes

**Fig. 6:**
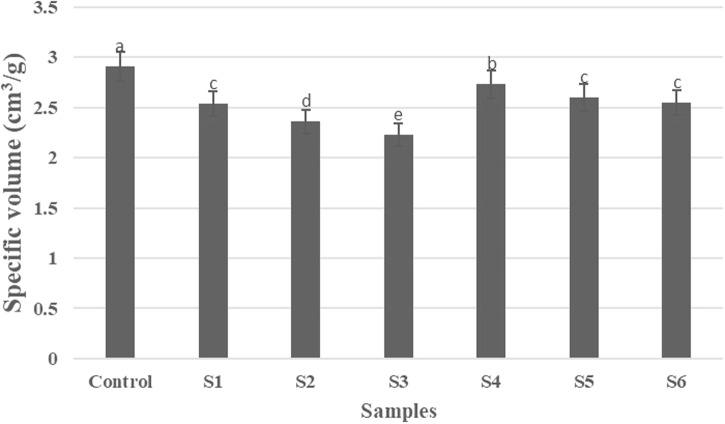
Specific volume of prepared cake samples.

As illustrated in Fig. 6, the control (100% wheat flour) recorded the highest specific volume (2.91 cm³/g), with significant changes compared to the other samples, reflecting a good ability to retain gases and ferment. The specific volume gradually decreased with increasing GCP content, reaching its lowest value at 30% substitution (2.23 cm³/g). This indicates a clear negative effect of GCP on the cake’s structure and air-holding capacity, possibly due to the higher fiber content or a change in protein properties.

**Fig. 7:**
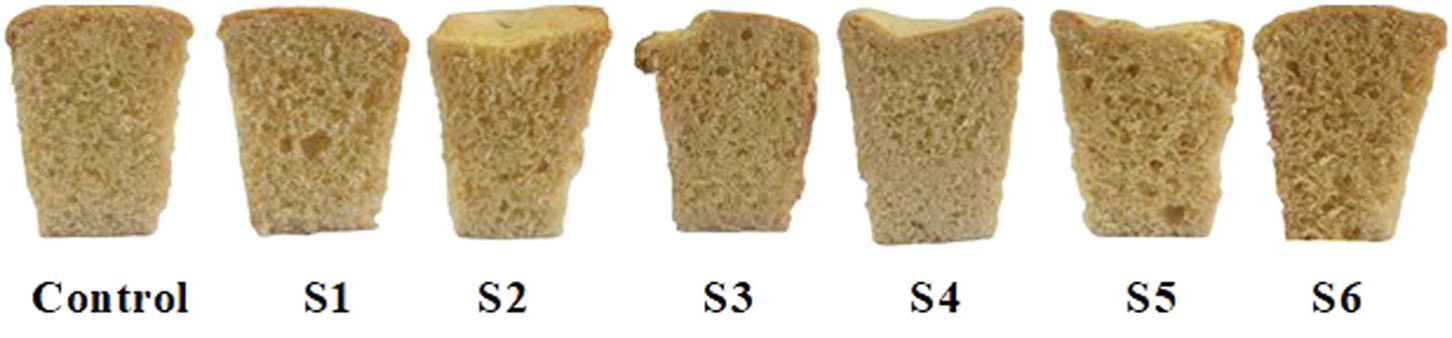
Photographs of prepared cake samples: Control cake prepared with 100% wheat flour. Samples S1–S3 were formulated by partially substituting wheat flour with germinated chickpea flour at levels of 10%, 20%, and 30%, respectively, while samples S4–S6 were formulated using germinated white kidney bean flour at the same substitution levels.

In the case of GWKB flour, the effect was less severe, as the samples retained relatively higher specific volumes compared to GCP-fortified cake, especially at the 10% GWKB substitution level (2.73 cm³/g), which was close to the control. With an increase in the substitution rate to 30% GWKB, the specific volume decreased to 2.55 cm³/g, but it remained superior to that of GCP-fortified cake at the same rate. These results reflect the possibility of substituting GWKB flour at moderate levels without significant loss in the quality of the cake’s airy texture, compared to GCP, which had a more pronounced effect in reducing the specific volume. The visual appearance of the prepared cake samples is shown in Fig. 7.

### Texture attributes of wheat flour cake and legume-fortified cakes

The texture profiles of the control cake (100% wheat flour) and legume-fortified cakes are presented in Table 6. Data stated that the control cake recorded the lowest hardness (10.25 N), reflecting a soft and friable texture. However, hardness gradually increased with increasing the replacement ratio of WF by GCP or GWKB, reaching its maximum hardness in the 70% WF + 30% GCP cake (22.17 N), followed by 16.89 N, which was recorded by the cake sample that was produced from 70% WF + 30% GWKP, indicating a firmer and denser texture. This is likely due to the higher fiber and protein content of the alternative flour and its effect on the gelatinous network structure of the cake^69^.

**Table 6:** Texture profile analysis of prepared cake samples

Cake samples	Texture parameters						
	H1 (N)	Resilience	H2 (N)	Cohesiveness	Springiness (cm)	Gumminess (N)	Chewiness (g.cm)
Control	10.25	0.13	8.35	0.33	0.94	3.42	328.0
S1	14.36	0.11	11.76	0.33	0.93	4.76	450.0
S2	17.13	0.11	13.64	0.32	0.95	5.43	528.0
S3	22.17	0.09	17.08	0.29	0.91	6.41	593.0
S4	14.07	0.10	11.38	0.31	0.94	4.37	417.0
S5	15.77	0.09	12.26	0.31	0.96	4.82	471.0
S6	16.89	0.09	14.20	0.32	0.92	5.46	509.0

H1: hardness cycle 1, H2: hardness cycle 2. The cake samples were formulated using wheat flour partially substituted with germinated legume flours. Chickpea flour, used in S1-S3 at substitution levels of 10%, 20%, and 30%, respectively, while white kidney bean flour, used in S4-S6 at the same levels, respectively. The control sample contained 100% wheat flour.

A slight decrease in resilience and springiness was observed in the legume-fortified cake samples compared to the control cake, indicating a loss of some of the rebound property after compression, especially in the GCP-fortified cake at high replacement ratios. Cohesiveness also decreased slightly in most of the legume-fortified samples, possibly reflecting a less cohesive structure compared to the control. Gumminess and chewiness values were higher for legume-fortified cake samples, particularly in the 70% GCP-fortified cake (gumminess: 6.41 N, chewiness: 593 g/cm), indicating a chewier texture, a characteristic that may be undesirable in baked products such as cakes. In contrast, the increase was less pronounced in the GWKB-fortified cake, indicating a relatively milder effect on texture compared to the GCP-fortified cake. In general, the incorporation of GCP flour into the cake formula as a wheat flour substitution has greater negative effects on cake firmness and chewiness than GWKB flour, especially at higher substitution ratios, which may be reflected in the sensory acceptance of the product. These results are in harmony with Bravo-Núñez and Gómez^63^, who reported that the addition of legume flour reduced cake volume and increased hardness in both sponge and layer cakes.

### Sensory attributes of prepared cake samples

Table 7 shows the effect of replacing wheat flour with different proportions of GCP and germinated white kidney beans (GWKB) on the sensory evaluation characteristics of cakes compared to a control cake sample (100% wheat flour, 72% ext.). The results showed that there was no significant difference (*p* > 0.05) between the control sample and the sample containing the lowest proportion of GWKB (10%) in terms of color and taste scores, while 20 and 30% GWKB-fortified cakes recorded a significant decrease in color and taste compared to the control sample. In general, the samples enriched with GWKB outperformed the samples enriched with GCP in terms of color, achieving significant differences between them at substitution ratios of 20 and 30%, while no significant changes were observed between them in the case of taste scores. The control sample also recorded the highest score of cake texture (8.78), with significant differences compared to the rest of the samples, as the lowest texture score (4.12) was observed in 30% GCP-fortified cake, compared to 4.46, which was observed in 30% GWKB-fortified cake, with a significant difference between them. Concerning the appearance of the cake, the sensory evaluation results indicated that there were no significant changes between the appearance of the control sample and 10% GCP-fortified cake or the 20 and 30% GWKB-fortified cakes, while the increase in the substitution ratios in both GCP and GWKB had a negative impact on the appearance of the resulting cake, with a significant difference compared to the appearance of the control sample. The control sample also significantly recorded the highest score for mouthfeel (8.70), followed by 8.14 for 10% GWKB-fortified cake, then 7.72 for 10% GCP-fortified cake, with significant differences between them.

**Table 7:** Sensory attributes

Cake samples	Sensory parameters						
	Color	Taste	Texture	Appearance	Flavor	Mouth feel	OAA
Control	8.48^a^ ± 0.43	8.50^a^ ± 0.47	8.78^a^ ± 0.21	8.42^a^ ± 0.37	8.44^a^ ± 0.29	8.70^a^ ± 0.18	8.50^a^ ± 0.09
**S1**	7.98^bc^ ± 0.23	8.02^b^ ± 0.14	7.76^bc^ ± 0.24	8.12^ab^ ± 0.25	8.10^b^ ± 0.22	7.72^c^ ± 0.29	7.96^b^ ± 0.16
**S2**	7.22 ^d^ ± 0.23	7.24^c^ ± 0.27	6.74 ^d^ ± 0.29	8.02^b^ ± 0.12	6.18^c^ ± 0.17	6.24 ^d^ ± 0.16	6.96^c^ ± 0.11
**S3**	4.60 ^f^ ± 0.35	5.72 ^d^ ± 0.39	4.12 ^f^ ± 0.35	7.50^c^ ± 0.22	4.28 ^d^ ± 0.24	3.80 ^f^ ± 0.22	5.04^e^ ± 0.08
**S4**	8.22^ab^ ± 0.29	8.30^ab^ ± 0.15	8.04^b^ ± 0.14	8.22^ab^ ± 0.19	8.38^ab^0.26±	8.14^b^ ± 0.16	8.26^a^ ± 0.08
**S5**	7.80^c^ ± 0.20	7.48^c^ ± 0.31	7.44^c^ ± 0.18	8.14^ab^ ± 0.15	6.10^c^ ± 0.13	6.32 ^d^ ± 0.12	7.22^c^ ± 0.04
**S6**	6.52^e^ ± 0.23	5.50 ^d^ ± 0.36	4.46^e^ ± 0.25	7.56^c^ ± 0.17	4.30 ^d^ ± 0.24	4.28^e^ ± 0.25	6.28 ^d^ ± 0.14
*p-*values	0.0011	0.0001	0.003	0.0001	0.001	0.0001	0.0001
LSD at 0.05	0.395	0.436	0.335	0.303	0.310	0.279	0.146

Values presented as means ± SD (*n* = 20). Different superscript letters (a, b, c, etc.) within the same column indicate significant differences (*p* < 0.05). The cake samples were formulated using wheat flour partially substituted with germinated legume flours. Chickpea flour, used in S1-S3 at substitution levels of 10%, 20%, and 30%, respectively, while white kidney bean flour, used in S4-S6 at the same levels, respectively. The control sample contained 100% wheat flour.

*OAA* overall acceptability.

In terms of overall acceptability, the control cake achieved the highest overall acceptability score (8.50), which falls within the “like very much” category, reflecting a very high palatability of the product. With the addition of GCP flour, a gradual decline in overall acceptability was observed; the score dropped to 7.96 at 10% GCP substitution (still in the “like moderately” category), but it declined further to 6.96 at 20% substitution (“like moderately”) and reached its lowest value at 30% substitution (5.04: “neither like nor dislike”), indicating that the cake’s sensory qualities were negatively affected as the percentage of GCP substitution increased.

For the GWKB, the results were relatively better at the 10% substitution level, where the cake maintained a high acceptability score (8.26), expressing “like very much,” with no significant changes from the control. Acceptability decreased to 7.22 at the 20% GWKB substitution level (in the “like moderately” category) and to 6.28 at 30% substitution (“like slightly”). These results demonstrate that substitutions of GCP flour and GWKB at moderate levels (up to 10% and 20%, respectively) remain sensorially acceptable and close to the control, while higher levels significantly decrease overall acceptability, necessitating a balance between increasing nutritional value and maintaining the taste and texture of the final product.

### Effects of phospholipase (ppm) and SSL (%) on crumb firmness and optimization based on response surface methodology

Although the fortification of bakery products with other grains, including legumes, improves the nutritional value of such products, it negatively impacts the freshness of the product, as fortified products are described as being fast stale (high crumb firmness) and thus less acceptable to consumers^70,71^. Therefore, the sample with the highest degree of hardness (based on texture profile analysis) was selected, and an attempt was conducted to reduce the crumb firmness of the cake sample using different concentrations of phospholipase (ppm) and SSL (%). Statistical analysis was performed using the response surface methodology to predict the optimum concentration that yielded the lowest crumb firmness. According to the obtained data, Table 8 represent the regression coefficients for the response surface model describing the effect of both phospholipase (ppm) and SSL (%) concentration on bread crumb hardness after 3 days of storage. The results show that both factors had a strong and direct significant effect (*p* < 0.0001), with an increase in either phospholipase or SSL concentration leading to a significant decrease in crumb hardness, reflecting their important role in improving bread softness. Both variables also showed significant quadratic effects, indicating that the effect is not entirely linear, but rather that there are optimum points for achieving the lowest possible crumb hardness.

**Table 8:** Regression coefficients of the response surface model for crumb firmness (N) after 3 days

Term	Coef	SE Coef	*T*-Value	*P*-Value	VIF
Constant	13.461	0.188	71.72	0.000	
Phospholipase (ppm)	−4.974	0.185	−26.95	0.000	1.00
SSL (%)	−3.238	0.185	−17.55	0.000	1.00
Phospholipase (ppm) × Phospholipase (ppm)	3.626	0.272	13.33	0.000	1.17
SSL (%) × SSL (%)	1.298	0.272	4.77	0.000	1.17
Phospholipase (ppm) × SSL (%)	0.057	0.226	0.25	0.800	1.00

This is confirmed by what is shown in Table 9, where the results of the analysis of variance (ANOVA) showed that the model as a whole was highly significant (*P* < 0.0001), and the model adequacy was confirmed by several statistical indicators. The non-significant lack-of-fit test (*P* = 0.357) demonstrated that the quadratic model adequately described the experimental data. The model also showed a very high coefficient of determination (*R*² = 95.74%), indicating that most of the variability in crumb firmness was explained by the studied factors. Examination of the residuals revealed no systematic patterns (Table 10), and the residuals were randomly and normally distributed around zero, confirming the validity and reliability of the fitted model. Data also indicated, the best conditions for achieving the lowest crumb firmness value were achieved when using 75.45 ppm of phospholipase with 0.60% SSL. These conditions are very close to achieving the pre-determined target (Target = 9.13 N) according to the optimization analysis. The Composite Desirability value reached approximately 0.961, reflecting a high agreement between the model and the desired target. The final Eq. (1) of the predictive model (the quadratic equation) is expressed as follows:

1 $${\rm{Crumbfirmness}}({\rm{N}})=26.654-0.2729\times {\rm{Phospholipase}}({\rm{ppm}})-19.64\times {\rm{SSL}}( \% )+0.001790\times {[{\rm{Phospholipase}}({\rm{ppm}})]}^{2}+14.42\times {[{\rm{SSL}}( \% )]}^{2}+0.0043\times {\rm{Phospholipase}}({\rm{ppm}})\times {\rm{SSL}}( \% )$$

**Table 9:** Analysis of variance (ANOVA) for the Model

Source	DF	Adj SS	Adj MS	*F*-Value	*P*-Value
Model	5	1354.24	270.848	265.12	0.000
Linear	2	1056.72	528.362	517.19	0.000
Phospholipase (ppm)	1	742.12	742.121	726.42	0.000
SSL (%)	1	314.60	314.604	307.95	0.000
Square	2	297.45	148.724	145.58	0.000
Phospholipase x Phospholipase	1	181.53	181.528	177.69	0.000
SSL x SSL	1	23.25	23.253	22.76	0.000
2-Way Interaction	1	0.07	0.066	0.06	0.800
Error	59	60.27	1.022		
Lack-of-Fit	3	3.35	1.117	1.10	0.357
Pure Error	56	56.92	1.016		
Total	64	1414.51

**Table 10:** Residual

Run	Observed (N)	Predicted (N)	Residual	Std. Residual
1	13.20	12.89	0.31	0.31
2	10.50	11.11	−0.61	−0.60
3	9.00	9.45	−0.45	−0.45
4	14.80	13.20	1.60	1.58
5	18.55	16.59	1.96	1.94

**Fig. 8:**
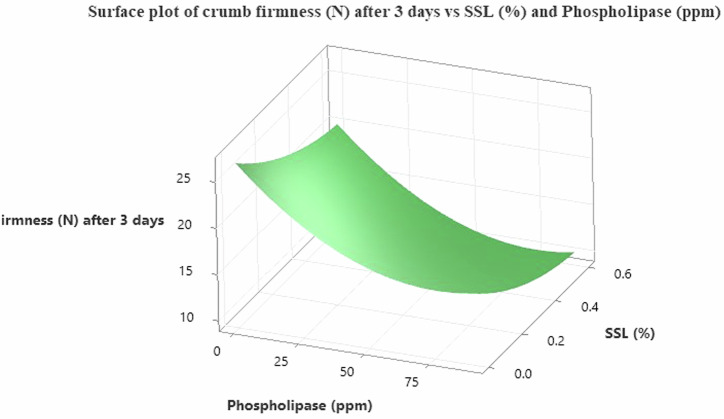
Surface plot of crumb firmness (N).

This is reinforced by Fig. 8, which illustrates the three-dimensional surface of the relationship between the studied factors. The figure shows a clear decrease in crumb hardness with increasing phospholipase concentration and increasing SSL ratio, confirming the synergistic effect of these two factors in improving product properties. However, the results showed that the binary interaction between phospholipase and SSL was not significant (*P* = 0.800). This indicates that each factor has its own independent effect on crumb toughness without a strong interaction between them. These results therefore underscore the importance of employing enzymes and surfactants such as SSL in improving bread quality, particularly in terms of softness and reducing crumb firmness during storage, thus achieving a more acceptable product for consumers. Several studies indicate that phospholipase-based improvers may offer advantages over amylase-type enzymes in bakery applications. Unlike amylases, which often retain excess moisture and may increase water activity, accelerating microbial spoilage^72,73^. Phospholipase generates lysophospholipids that function as natural emulsifiers, enhancing crumb softness while limiting water redistribution during storage. This mechanism contributes to improved freshness and reduced staling without elevating water activity with softer crumbs and better shelf-life stability compared to traditional enzymatic improvers^74,75^.

## Methods

### Samples and chemicals

Medium-strength flour (12.89% moisture, 0.52% ash, 10.65% protein, 1.11% lipids, 0.41% crude fiber, and 87.91% total carbohydrates) was obtained from Amoun for Milling Company, Giza, Egypt. Chickpea (*Cicer arietinum* L) and white kidney bean (*Phaseolus vulgaris* L) were purchased from the local market, Giza, Egypt. The Grindsted sodium stearoyl lactylate (code: P 2522) emulsifier, Danisco Co. (Cotia, SP., Brazil), and phospholipase A1 (EC 3.1.1.32) Lipopan® F (18.5 KLU/g, Novozymes A/S, Denmark) were obtained from Chemitec Co., Alexandria, Egypt. All chemicals and reagents utilized in this research were of analytical grade and high purity.

### Germination process

Seeds were manually cleaned to remove dust, stones, and damaged kernels. The cleaned chickpea and white kidney bean seeds were soaked in distilled water for 8 h at room temperature, then surface-sterilized using 1% sodium hypochlorite for 10 min and rinsed thoroughly with distilled water. The sterilized seeds were transferred to sterile Petri dishes lined with moistened filter paper and germinated for 72 h at 37 ± 2 °C, with periodic spraying to maintain moisture. Sprouted seeds were finally rinsed with distilled water and immediately subjected to further processing.

### Preparation of germinated legume flour

The germinated seeds were frozen at −40 °C ± 2 and then lyophilized for 72 h in a freeze-dryer (LABCONCO, Kansas, USA) at −50 °C and 0.014 mbar to reaching a moisture level of <5.0%. Dry materials were mill in a stainless steel mill (GRINDER, KA3025, China) and then sieved through a 100-μm sieve. The fine materials were stored in sealed polyethylene bags under refrigeration at 5 °C ± 1 until use.

### Cake preparation

Cakes were prepared according to Ertaş^76^ with some modifications: 100 g flour, 72 g shortening, 88 g sugar, 80 g whole egg, 1.6 g vanilla, 8.0 g skimmed milk powder, 2.4 g salt, and 3.0 g baking powder. The control cake was produced with 100% wheat flour, and the formulations of legume-fortified cakes are mentioned in detail in Table 11. As for the cake preparation, shortening, eggs, and sugar were mixed in a mixer (Kenwood KVL4230S, China) for two minutes at low speed. The rest of the components were added after mixing, and the mixture was once more blended for one minute on a lower speed and two minutes on a medium speed. The batter was poured into 8 × 5 × 12 cm molds and baked for 35 min at 175 °C. Samples were cooled to room temperature and stored in polyethylene bags until use.

**Table 11:** Cake formulas

Ingredients (gm)	Control	Blends					
		S 1	S 2	S 3	S 4	S 5	S 6
**Wheat Flour**	100.0	90.0	80.0	70.0	90.0	80.0	70.0
**Germinated chickpea flour**	-	10.0	20.0	30.0	-	-	-
**Germinated white kidney beans flour**	-	-	-	-	10.0	20.0	30.0
**Skimmed milk**	8.0	8.0	8.0	8.0	8.0	8.0	8.0
**Sugar**	88.0	88.0	88.0	88.0	88.0	88.0	88.0
**Fresh egg**	80.0	80.0	80.0	80.0	80.0	80.0	80.0
**Shortening**	72.0	72.0	72.0	72.0	72.0	72.0	72.0
**Salt**	1.5	1.5	1.5	1.5	1.5	1.5	1.5
**Vanilla powder**	1.6	1.6	1.6	1.6	1.6	1.6	1.6
**Baking powder**	3.0	3.0	3.0	3.0	3.0	3.0	3.0

### Chemical analysis

The moisture content was estimated in accordance with the AACC, 44-19 technique (135 °C for 2.5 h). Ash content was estimated according to AACC, 08–01 at 550 °C. According to the AACC, the 30–25 procedure, crude fat was extracted using n-hexane by oil extraction equipment (Velp SER 148/3, Usmate, Italy). The Kjeldahl method is used to estimate crude protein (AACC 46-12). Every estimate is done using dry methods^77^. Differences were used to determine NFE according to Singer et al.^78^, as following: 100- (protein, % + ash, % + fat, % + fiber %). All analyses were performed in triplicate. Results are expressed as mean ± standard deviation.

### Minerals determination

The mineral content (Fe, Zn, K, Mg, and Ca) was determined by inductively coupled plasma atomic emission spectrometry (ICP—AES: Varian, Vista, Zug, Switzerland) according to Skujins^79^. All analyses were performed in triplicate. Results are expressed as mean ± standard deviation.

### Functional properties

On a dry base, the water absorption capacity (WAC) of the fine flour was estimated according to the AACC method 56–20^77^. The oil absorption capacity (OAC) of the flours was assessed according Setia et al.^56^. Foaming capacity (FC) of the samples was estimated according to Makri and Doxastakis^80^ at room temperature, after 30 s, FC was represented as the volume increase as a percentage. Emulsification capacity (EC) was determined at room temperature according Naczk et al.^81^, for 30 s, 1.0 g of the material was mixed with DW (50 ml) until the emulsion breakpoint was attained, constantly, corn oil was added from a buret while blending the mixture. The total amount of oil was expressed as the emulsifying capacity. All analyses were performed in triplicate. Results are expressed as mean ± standard deviation.

### Pasting properties

RheoLab QC (Anton Paar, Graz, Austria) was calibrated to measure the pasting behavior of wheat flour compared to germinated and ungerminated legume flour (14% moisture basis) for determining the pasting properties. After 1 min of equilibration at 50.0 °C, the legume flour was heated to 95.0 °C (rate: 12.0 °C/min), maintained for 2.5 min, cooled to 50.0 °C (rate: 12.0 °C/min), and then held at 50.0 °C for two minutes once more. Throughout the analysis, a continuous paddle rotating at 160 rpm was employed. The analysis was performed in duplicate. Results are expressed as mean ± standard deviation.

### Determination of tannins content

Two grams of the sample were extracted using 10 ml of a one percent HCl solution in methanol for 24 h while being continuously shaken, and the sample was centrifuged for 5.0 min at 1000 rpm. After 20.0 min, the absorbance was measured at 500 nm using 1.0 ml of the supernatant and 5.0 ml of vanillin-HCl reagent, which is made up of equal parts 8.0% HCl and 4.0% vanillin in methanol. Condensed tannins were quantitatively determined using D-catechin as a reference solution^82^. The analysis was performed in duplicate. Results are expressed as mean ± standard deviation.

### Determination of phytic acid content

Young and Greaves’s^83^ standard method was applied for phytic acid determination. After being soaked for 3 h in 100 ml of 2% HCl acid, 4 g of the sample were filtered. A brownish yellow tint persisted for 5 s. after 25 ml of filtrate, 5.0 ml of 0.30% NH_4_SCN, and 53.0 ml of DW were combined and titrated against a 0.01 M standard FeCl_3_ upon color change (1.0 mL = 1.19 mg phytin phosphorous), the phytic acid content was computed by multiplying the phytin phosphorus value by 3.55 as reported by Krishna and Ranjhan^84^. The analysis was performed in duplicate.

### Protein digestibility

The in vitro protein digestibility (IVPD) of ground legumes was estimated using a pepsin digestion assay under acidic conditions. According to Hsu et al.^85^, briefly, approximately 0.50 g of sample was weighed into filter bags and incubated in a digestion solution containing HCl (0.1 M) and pepsin enzyme (1 g/L) to simulate gastric conditions. Samples were incubated in a DaisyII incubator (ANKOM Technology, USA) at 39 ± 1 °C for 24 h. Following incubation, the filter bags were removed and placed in a water bath for 30.0 minutes at 80.0 °C ± 2 to terminate enzymatic activity. Bags were washed by DW three times to remove soluble residues, and subsequently dried at 105.0 ± 1.0 °C in a hot air oven for 24 h to determine the dry weight of the undigested residues. The total nitrogen of the undigested residues was assessed using the mini-Kjeldahl. The percentage of IVPD was calculated as the following equation:

2 $${IVPD}\,\left( \% \right)=\left[\frac{\left({Total}\,{protein}-{undigested}\,{protein}\right)}{\left({Total}\,{protein}\right)}\right]* 100$$

### Preparation of extract

The method started with a mix of 1.0 g of the sample with methanol (80.0%), at a total volume of 20.0 ml, with an agitation rate of 250 rpm at room temperature for 24 h. The suspension centrifuged for 10.0 min (10,000 rpm), at 4.0 °C (Hettich, Mikro R22, Germany), and then the supernatant filtered using a PVDF filter (0.45 µm) and refrigerated (4.0 °C ± 1.0) until its analysis^86^.

### Total phenolic

Folin & Ciocalteu’s phenol (FCP) reagent was used in a colorimetric approach to measure the TPC in legume extract^87^. In short, 0.25 ml extract solutions were combined with 4.0 ml of water, 0.50 ml of Na_2_CO_3_ (aqueous saturated solution), and 0.25 ml of the FCP reagent. The samples were centrifuged for 5.0 min at 5000 × *g* using a Sigma 3–18KS centrifuge (SIGMA, Laborzentrfugen, Osterode, Germany) after standing in the dark for twenty-five minutes. Supernatants’ absorbance was measured at 725 nm using a JASCO V-730 spectrophotometer (Japan, Tokyo, Corp.). TPC expressed as milligrams of GAE per gram. The analysis was performed in triplicate, and the results are expressed as the mean.

### DPPH antioxidant activity

A 50 μL of extract and 950 μL of DPPH methanolic solution (100 µM) were incubated in a dark area for 15 min at room temperature to measuring at 517 nm using a JASCO V-730 spectrophotometer (Japan, Tokyo, Corp.). The activity for the elimination of DPPH radicals was expressed as Trolox millimole equivalent per gram (mmol ET g^−1^) of the dry sample^88^. The analysis was performed in triplicate, and the results are expressed as the mean.

### ABTS antioxidant activity

The activity for the elimination of ABTS°+ radicals was estimated in accordance to Re et al.^89^, by dissolving the ABTS in DW at a concentration of 7.0 mM, by causing the ABTS solution to react with potassium persulfate, the radical was formed at 2.45 mM, and the prepared mix was left to rest at room temperature for 16 h in a dark area. The solution of ABTS°+ radicals was diluted with ethyl alcohol until an absorbency of 0.70 was reached at 734 nm (JASCO V-730 spectrophotometer, Japan, Tokyo, Corp.). The mix was 10 µL of extract with 990 µL of ABTS°+ radical, and the reaction time was a 15 min period, where at the end an absorbency reading was done. Millimole trolox equivalent per gram of the dry material (mmol ET g^−1^) was used to express the results. The analysis was performed in triplicate, and the results are expressed as the mean.

### Amino acids profile

Amino acids were estimated after hydrolysis of the defatted samples as well as its formulated samples with 6.0 N HCl, at 110 °C for 22 h in a nitrogen atmosphere using a Beckman amino acid analyzer (Model 118/119 CL) according to Moore and Stein^90^. Protein efficiency ratio (C-PER) was calculated as explained by Alsmeyer et al.^91^.: C-PER is equal to −0.047 (proline) + −0.684 + 0.456 (leucine). Biological value (BV) calculated as 49.9 + (10.53*C-PER) as described by Yan et al.^92^, Fischer value calculated as a mole ratio of BCAAs (valine, leucine, and isoleucine) over total aromatic amino acids (tyrosine and phenylalanine).

### Color measurement

A Chroma Meter CR 400 (Minolta, Japan) used for measure the color attributes. According CIELAB system, L* values ranged from 0 (black) to 100 (white), a* values indicates green (-60) to (+60) red, and b* values indicates blue (−60) to yellow (+60). Chroma and hue were calculated according to the Eqs. 3 and 4. The browning index (BI) was calculated according to Eq. five^93^. According to Bilgen et al.^94^, the whiteness index (WI) was calculated as presented in Eq. 6.

3 $${Hue}=\arctan (\frac{{a}^{* }}{{b}^{* }})$$

4 $${Chroma}={[{\left({a}^{* }\right)}^{2}+{\left({b}^{* }\right)}^{2}]}^{1/2}$$

5 $${BI}=\frac{100(x-0.31)}{0.172}$$

$${Where}:\,x=\frac{({a}^{* }+1.75{L}^{* })}{{(5.645L}^{* }\,+\,{a}^{* }\,-\,3.012{b}^{* })}$$

6 $${WI}=100-\sqrt{{(100-{L}^{* })}^{2}+{{(a}^{* })}^{2}{{(b}^{* })}^{2}}$$

### Specific volume

The samples were cut to a suitable size for measurement, weighed, and the volume was estimated by displacement of the rapeseed. The volume was divided by the weight to obtain the specific volume expressed in cm^3^/g^95^. The analysis was performed in triplicate. Results are expressed as the mean.

### Instrumental textural profile analysis (TPA)

The texture analysis (TPA) was performed for cake samples using a CT3 Texture Analyzer equipped with TexturePro CT V1.6 software (Brookfield Eng., Lab., Middleboro, MA, USA). A cylindrical aluminum probe (TA-AACC36) and a 10.0 kg load cell were used under the following conditions: test speed 2.0 mm/s, two compression cycles, and 50% strain. TPA parameters were automatically calculated by the software^96^.

### Sensory evaluation

Twenty healthy judges aged 25–50 years voluntarily participated in the sensory evaluation after providing informed consent. Each panelist received a sensory evaluation form and approximately 25 g of each cake formulation. A 9-point hedonic scale was used to evaluate appearance, color, taste, texture, flavor, and overall acceptability (1 = extremely dislike, 9 = extremely like). Water was provided between samples to cleanse the palate and minimize carry-over effects^97^. All procedures involving human participants were conducted in accordance with the ethical standards of the institutional research committee and the Declaration of Helsinki (1964) and its later amendments. The sensory evaluation protocol was reviewed and approved by the Sensory Evaluation Ethics Committee (SEC-18), Food Technology Research Institute, Agricultural Research Center (ARC), Egypt. Written informed consent was obtained from all panelists prior to participation, and participants were informed of the nature of the samples and their right to withdraw at any time without penalty.

### Improvement of crumb cake freshness

The sample with the highest degree of hardness (based on texture profile analysis) was selected to improve their freshness by phospholipase (ppm) and SSL (%) at different levels to evaluate the crumb firmness after 72 h of storage. A design matrix was applied in five replications as presented in Table 12.

**Table 12:** Central composite design of phospholipase and SSL as cake improvers

Point Type	Coded A	Coded B	Phospholipase (ppm)	SSL (%)	Replicates (n)
Factorial	−1	−1	0	0.0	5
Factorial	−1	+1	0	0.6	5
Factorial	+1	−1	90	0.0	5
Factorial	+1	+1	90	0.6	5
Axial (face)	0	−1	45	0.0	5
Axial (face)	0	+1	45	0.6	5
Axial (face)	−1	0	0	0.3	5
Axial (face)	+1	0	90	0.3	5
Center	0	0	45	0.3	25

### Firmness measurement

AACC Method 74-09.01 was used to test the crumb’s firmness after 72 h of storage^26^. Cake firmness was measured using a CT3 Texture Analyzer (Brookfield Eng., Lab., Middleboro, MA, USA) fitted with a TA-AACC36 (cylindrical aluminum probe) and a 10.0 kg load cell. Uniform slices (5.0 × 5.0 × 2.5 cm) were compressed once to 50% of their height at a speed of 1 mm/s. The maximum force recorded during compression was considered as the firmness value.

### Statistical analysis

One-way ANOVA was performed to assess differences among group means (*p* < 0.05) in three replicates. A central composite design (Table 12) was created and analyzed by Minitab Statistical Software (Version 22, Minitab LLC, USA) to study the effects of phospholipase (ppm) and SSL (%) on crumb firmness after 3 days. Data were fitted to a quadratic model, and ANOVA was performed to test significance at a 95% confidence level. Model adequacy was checked by lack-of-fit tests and residual analysis.

## Data Availability

The datasets generated in the current study are available from the corresponding author on reasonable request.

## Code availability

No custom code was generated or used in this study.
